# Prevalence of Acute Kidney Injury in Patients with Liver Cirrhosis

**DOI:** 10.31729/jnma.5147

**Published:** 2020-08-31

**Authors:** Pukar Thapa, Sudhamshu KC, Achyut Bikram Hamal, Dilip Sharma, Sandip Khadka, Niyanta Karki, Bikash Jaishi, Pratap Sagar Tiwari, Anshu Vaidya, Binod Karki

**Affiliations:** 1 Liver Unit, Department of Medicine, National Academy of Medical Sciences, Bir Hospital, Kathmandu, Nepal; 2 Department of Medicine, Nepal Army Institute of Health Sciences, Kathmandu, Nepal

**Keywords:** *acute kidney injury*, *hepatorenal syndrome*, *liver cirrhosis*

## Abstract

**Introduction:**

Acute kidney injury is a common and life-threatening event in patients with liver cirrhosis occurring in approximately 20-50% of hospitalized patients of liver cirrhosis. Pre-renal acute kidney injury, the hepatorenal syndrome type of acute kidney injury and acute tubular necrosis represent the common causes. The aim of this study was to study the profile of acute kidney injury in patients with liver cirrhosis.

**Methods:**

Consecutive patients of liver cirrhosis admitted in Liver unit of Bir Hospital were studied to see the presence of acute kidney injury in this hospital based descriptive cross-sectional study. Clinical and laboratory parameters along with various clinical outcome were compared between different groups categorized by the severity of liver disease and renal dysfunction.

**Results:**

Out of 302 liver cirrhosis patients, 56 (18.5%) had acute kidney injury among which 23 (46%) were found to have pre-renal acute kidney injury, 15 (30%) with hepatorenal syndrome-acute kidney injury and 12 (24%) with intrinsic renal disease. Patients with higher stages of acute kidney injury had longer duration of hospital stay and hepatorenal syndrome-acute kidney injury was seen in patients with higher grade of ascites and with hyponatremia.

**Conclusion:**

Acute kidney injury is a common occurrence in patients with advanced liver cirrhosis with pre-renal acute kidney injury being the commonest cause. Median hospital stay is directly affected by the severity of acute kidney injury and hepatorenal syndrome-acute kidney injury was seen in patients with higher grade of ascites and hyponatremia. Early identification of patients at high risk for acute kidney injury may help to reduce mortality and contain costs.

## INTRODUCTION

Kidney dysfunction is a common and life-threatening event in patients with liver cirrhosis (LC). Acute kidney injury (AKI) has an estimated prevalence of 20-50% among hospitalized patients with LC.^1,2^ Parameters of renal dysfunction are powerful predictors of death in decompensated LC^3^ and is reflected by the inclusion of serum creatinine (sCr) in the Model for End Stage Liver Disease (MELD) Score, which is used for assessment of severity of liver disease and prioritization of patients with advanced liver disease for liver transplantation.^4^

Pre-renal AKI, the hepatorenal syndrome type of AKI (HRS-AKI) and acute tubular necrosis represent the common causes of AKI in LC. There are very few studies documenting AKI in patients with LC admitted in the hospital.

The aim of this study was to find the prevalence of AKI in patients with LC and to see the clinical outcome of the patients in various stages of acute kidney injury and severity of liver disease.

## METHODS

This was a hospital based descriptive cross-sectional study carried out in a tertiary hospital of Kathmandu. Consecutive patients of LC who were 18 years and above and willing to participate, admitted in the liver unit of Bir hospital were included in the study to see the presence of renal impairment. Those patients with AKI were analysed. Patients with post liver transplantation status, associated with other serious diseases affecting the mortality such as heart failure or coronary artery disease, presence of diabetes and hypertension, diagnosed with primary (hepatocellular carcinoma or chol-angiocarcinoma) or metastatic malignancies, patients with primary kidney disease and chronic kidney disease were excluded.

Sample size was calculated as:

n = Z^2^ p (1-p)/e^2^

where,

Z = confidence level at 95% (standard value of 1.96)
P = estimated prevalence or proportions of projected area
e = range of CI

Now substituting p = 22% from previous study^2^ and e = 5%, we get the value of 264.

Since, we have finite population and expected case per year is 60.

Sample size for finite population is:

Sn = n × N/ (n + N - 1)

where N is expected case per year = 60

Hence final sample size is 50.

Clinical evaluation for all patients was done, including history, clinical examination, and investigations that included liver and renal function tests, complete blood count and coagulation profile. Six patients were not included in the study because 5 did not meet the inclusion criteria and one refused to participate.

LC was diagnosed on the basis of clinical evaluation, liver function tests and imaging diagnosis with or without liver biopsy.^5^ Diagnosis and grading of renal dysfunction along with diagnosis of HRS-AKI was made according to The Revised Consensus Recommendations of the International Ascites Club on the Diagnosis of Acute Kidney Injury in Liver cirrhosis.^6^ Proportion of renal dysfunction was calculated as percentage of each category. Background information, types of AKI and aetiology of LC was calculated according to their frequencies. Laboratory parameters of the patients on admission and discharge was compared. Relationship of grading of AKI was seen with clinical outcome and duration of the hospital stay of the patients. Types of AKI was compared with severity of liver disease as reflected by Child Pugh class and MELD score. Relationship of HRS-AKI was seen with grades of ascites and levels of sodium. Ethical approval was taken from the institutional review board of the institute. Information collected from the patients were entered and analysed using SPSS 20 software. Continuous data were expressed as mean (SD), and a categorical variable as number (%).

## RESULTS

**Figure 1.**
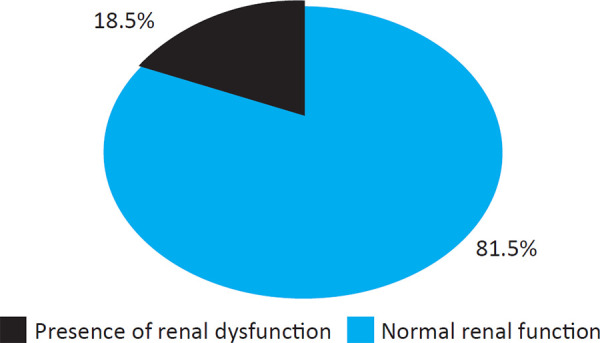
LC patients by renal function.

During study period, 302 patients with LC were screened. Fifty six (18.5%) patients had AKI. Among fifty six patients with AKI, six patients were excluded from the analysis due to not meeting the inclusion criteria (Figure 1).

**Figure 2.**
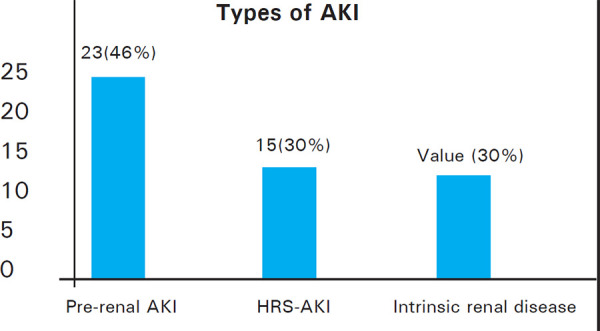
LC patients by types of AKI.

Out of 50 patients with AKI, 23 (46%) were found to have pre-renal AKI, 15 (30%) with HRS-AKI and 12 (24%) with intrinsic renal disease (Figure 2).

Mean age of the patients was 51.82 years with SD of 11.11. Males were slightly more in number in comparison to females 27 (54%) vs 23 (46%). Most common cause of LC was alcohol accounting to 41 (82%). Other causes of LC were HBV related 1 (2%),NASH related 2 (4%)and mixed 6 (12%).

Infection was common finding present in 20 patients (40%). The most common infection was UTI in 17 (34%) followed by SBP in 7 (14%)and few of them having multiple site infections. Ascites was present in almost all of the cases 47 (94%). Thirty-one patients (62%) had moderate ascites, while 14 (28%) and 2 (4%) had severe and mild ascites respectively. Hepatic encephalopathy and upper GI bleed was present in 16 (32%) and 15 (30%)of the patients respectively and almost all the patients were on diuretics.

Laboratory parameters of the patients on admission and discharge are shown (Table 1). Though other lab values remained grossly similar, there was difference seen in peripheral WBC, sodium, urea and creatinine between admission and discharge.

**Table 1:** Laboratory parameters of the patients on admission and discharge n = 50.

Clinical parameters	Day of admission Mean (± SD)	Day of discharge/death Mean (± SD)
Hemoglobin (g/dl)	8.9 (1.7)	8.9 (1.3)
WBC (cells/mm^3^)	12770 (7064)	8432 (4490)
Plateletes (cells/mm^3^)	105210 (67877)	93740 (49027)
Urea (mg/dl)	84.9 (32.2)	48.4 (23.2)
Sodium (mmol/L)	130.4 (6.7)	131.4 (6.5)
Potassium (mEq/L)	3.7 (0.3)	3.8 (0.1)
Bilirubin (mg/dl)	7.0 (6.5)	6.9 (5.8)
Albumin (g/dl)	2.4 (0.5)	2.9 (2.6)
INR	1.8 (0.3)	1.7 (0.2)
Creatinine (mg/dl)	2.6 (1.2)	1.2 (1.1)

Twenty one (42%) of the patients had stage 1 AKI while 14 (28%) of the patients had stage 2 and 15 (30%) had stage 3 AKI. Forty six (92%) of the patients improved and was discharged while only 4 (8%) of the patients died. Two of the patients who died, developed sepsis with septic shock, one had massive upper GI bleeding and one developed hepatic encephalopathy grade IV. Dialysis was required in 3 patients among which 2 died and one survived. The patients who underwent dialysis were both diagnosed with HRS-AKI while the one who survived was diagnosed to have intrinsic renal disease. This study showed that the patient who presented with stage 1 and stage 2 AKI had shorter stay in the hospital while those who presented with stage 3 AKI had a longer stayas shown (Table 2).

**Table 2:** Relationship of staging of AKI with clinical outcome and duration of hospital stay of the patients.

Grading of AKI	Clinical outcome		Duration of hospital stay		
	Improved	Death	< 1 week	1-2 weeks	>2 weeks
**Stage 1**	20 (40)	1 (2)	8 (16)	10 (20)	3 (6)
**Stage 2**	13 (26)	1 (2)	5 (10)	7 (14)	2 (4)
**Stage 3**	13 (26)	2 (4)	1 (2)	4 (8)	10 (20)
**Total**	46 (92)	4 (8)	14 (28)	21 (42)	15 (30)

All the types of AKI were more common in patients with Child Pugh C class 46 (92%)and almost half of the patients 22 (44%) were having a MELD score between 18-25 as shown (Table 3).

**Table 3:** Relationship of types of AKI with severity of liver disease.

Variable	Child Pugh B	Child Pugh C	MELD (<18)	MELD (18-25)	MELD (>25)
PRE-RENAL	1 (2)	22 (44)	7 (14)	8 (16)	8 (16)
AKI-HRS	1 (2)	14 (28)	6 (12)	7 (14)	2 (4)
INTRINSIC	2 (4)	10 (20)	3 (6)	7 (14)	2 (4)
TOTAL	4 (8)	46 (92)	16 (32)	22 (44)	12 (24)

Fifteen (30%) of the patients were diagnosed as HRS-AKI and remaining 35 (70%) of the patients were diagnosed to have other types of AKI (pre-renal and intrinsic renal disease). HRS-AKI was seen more commonly in advanced grade of ascites as categorized by mild, moderate and severe which was not consistent with non-HRS patients. Also, HRS-AKI was seen in the patients with lower level of sodium in comparison with non-HRS patients.

**Table 4:** Relationship of HRS with ascites and hyponatremia.

	Ascites				Sodium		
	Absent	Mild	Moderate	Severe	<126	126-135	>135
Non HRS AKI	3 (6)	2 (4)	24 (48)	6 (12)	0 (0)	21(42)	14 (28)
HRS-AKI	0 (0)	0 (0)	7 (14)	8 (16)	13 (26)	1 (2)	1 (2)
Total	3 (6)	2 (4)	31 (62)	14 (28)	13 (26)	22 (44)	15 (30)

## DISCUSSION

This study showed that 56 (18.5%) of the patients with LC admitted in the hospital had AKI. Pre-renal AKI, HRS-AKI and AKI due to intrinsic renal disease were the spectrum seen in this studyand pre-renal was the most common form of AKI 23 (46%). AKI has an estimated prevalence of approximately 20-50% among hospitalized patients with LC in various other studies^2,7,8^ and is associated with poor prognosis and represents an important predictor for short-term mortality in patients with LC.^9,10^ The lower prevalence of AKI in our study could be due to delay in the diagnosis of AKI due to various reasons.

Mean age of the patients in this study was 51.8 years and alcohol was the most common etiology of LC 41 (82%). This finding was similar with the study done by Mohan J et al in Chennai where he found out that mean age was 48 and 85% of them had alcohol as the etiology of LC. Since alcohol consumption is common among many ethnic group of Nepal and also supported by various cultures and beliefs, it has become the major cause of LC in our society. Contrary to our study, where males and females were almost equally affected 27 (54%) vs 23 (46%), there was male predominance with only 5% of female showing AKI in a study done by Mohan J et al. This difference in the finding that almost half of the patients of this study were female is possibly due to the cultural differences and increasing alcoholism among females owing to decline in social stigmata attached to drinking and to the ready availability of alcohol.

Acute kidney injury, defined by a significant reduction in glomerular filtration rate (GFR) over a short time period, is often triggered by a precipitating event (i.e. overdose of diuretics, large-volume paracentesis without albumin replacement, gastrointestinal bleeding, bacterial infections, etc.)^11^ AKI has been documented to occur mostly secondary to hypovolemia (gastrointestinal hemorrhage, aggressive diuresis, or diarrhea), infection and drugs in various other studies.^12^ In this study as well, infection and upper GI bleed was present in 20 (40%) and 15 (30%) of the patients respectively. Upper GI bleed was more common in pre-renal AKI and the most common infection was UTI followed by SBP. In this study, 16 (32%) of the patients had hepatic encephalopathy of any grade and almost all the patients were using diuretics. It has been documented that diuretics were associated with the risk of renal injury and factor such as hepatic encephalopathy has been associated with the progression of AKI.^13^ The limitation of this study is being unable to perform urine sodium concentrations, fractional excretion of sodium (FENa) and kidney biopsy to diagnose acute tubular necrosis in these patients. Hence, patients were diagnosed with intrinsic renal disease on basis of their clinical characteristics and laboratory parameters after excluding pre-renal AKI, HRS-AKI and post-renal AKI.

There was a significant drop in the renal function test and peripheral WBC at discharge compared to the admission in the patients in this study. Also, there was significant improvement of sodium level in these patients. Since infection was one of the common precipitant of AKI in LC, this finding can be attributed to early use of antibiotics for UTI and SBP, and also timely correction of hyponatremia that was present in our patients. Few other studies also showed positive correlation between CRP, TLC with serum creatinine which indicates that infection has a role in developing AKI.^14^

In this study, the mortality was seen in 4 patients (8%). Mortality was more in patients with stage 3 AKI compared to stage 1 and stage 2 (1 patient in each stage). In one prospective study on 192 hospitalized patients with LC, in-hospital mortality varied from 2% for AKI stage 1, 7% for stage 2, and up to 21% for stage 3. Furthermore, the mortality rates were 29% and 60% for stage 1 patients progressing to stage 2 and 3, respectively. Mortality increased to 19% for patients presenting in stage 2 and then progressing to stage 3 during the hospital stay. Patients with endstage renal disease requiring dialysis had 60-71% in-hospital mortality.^15^ Another study also showed that increasing severity of AKI is strongly associated with inhospital mortality.^16^ In this study, increasing mortality was seen as the stages of AKI progressed. This finding is probably related to lower number of mortality in this study attributing to mortality taken only during hospital stay. Duration of hospital stay was also longer in stage 3 compared to stage 1 and stage 2. The prognosis for patients with LC and AKI is very poor and it gets worse for advanced stages of liver disease as well as AKI.^17,18^ Also, increased duration of renal failure prior to transplant appears to be a negative predictor of post-transplant renal function.^19^ Almost all the patients with Child Pugh C (92%) developed AKI in this study.

Dilutional hyponatremia and HRS represent manifestations of a continuum of pathophysiologic events stemming from portal hypertension and the resultant vasodilatation, which are the main mechanisms responsible for the development of ascites. In a prospective inception cohort study of patients with LC and new-onset ascites who were followed for a mean of 41 months, hyponatremia developed in 28% of the patients, 11% developed refractory ascites, and 8% developed HRS, suggesting a sequential process (from ascites to hyponatremia to refractory ascites to HRS).^20^ In this study as well, HRS-AKI was more common in patients with increasing grade of ascites reflecting that worsening of ascites is directly proportional to development of HRS. Also HRS-AKI was far more common in patients who had lower sodium level in comparison to patients with higher sodium level in blood.

## CONCLUSIONS

In conclusion, AKI is a common occurrence in patients with advanced LC. Pre-renal AKI is the commonest cause among various causes of AKI. Median hospital stay and cost is directly affected by the severity of AKI as well as liver disease. Early identification of patients at high risk for AKI in LC along with early diagnosis and treatment is imperative for improving outcomes and reducing cost. Hyponatremia and higher grades of ascites is directly related to HRS in a cirrhotic patients. Early diagnosis and treatment of these complications of LC may help to reduce the mortality and morbidity in such patients.

## Conflict of Interest

**None.**
